# Computation and Simulation of Evolutionary Game Dynamics in Finite Populations

**DOI:** 10.1038/s41598-019-43102-z

**Published:** 2019-05-06

**Authors:** Laura Hindersin, Bin Wu, Arne Traulsen, Julian García

**Affiliations:** 1 Department of Evolutionary Theory, Max Planck Institute for Evolutionary Biology, Plön, Germany; 2 School of Science, Beijing University of Posts and Telecommunications, Beijing, China; 3 Faculty of Information Technology, Monash University, Melbourne, Australia

**Keywords:** Computational models, Evolutionary theory

## Abstract

The study of evolutionary dynamics increasingly relies on computational methods, as more and more cases outside the range of analytical tractability are explored. The computational methods for simulation and numerical approximation of the relevant quantities are diverging without being compared for accuracy and performance. We thoroughly investigate these algorithms in order to propose a reliable standard. For expositional clarity we focus on symmetric 2 × 2 games leading to one-dimensional processes, noting that extensions can be straightforward and lessons will often carry over to more complex cases. We provide time-complexity analysis and systematically compare three families of methods to compute fixation probabilities, fixation times and long-term stationary distributions for the popular Moran process. We provide efficient implementations that substantially improve wall times over naive or immediate implementations. Implications are also discussed for the Wright-Fisher process, as well as structured populations and multiple types.

## Introduction

Theoretical models of evolutionary games in finite populations typically require numerical procedures or simulations^1–5^. This is even the case when analytical results exist, as these are often difficult to interpret or confined to specific limits^6–13^. Simulations as well as numerical approximations are therefore common in the field, but far from being standardised. There are different computational methods to assess the key quantities in evolutionary game dynamics. Here we focus on studying the popular Moran process^6^. The purpose of this paper is to give an overview of such computational methods and to compare their limitations and scalability. We provide algorithms in pseudo-code as well as the source code for all the procedures that we study.

The Moran process^14^ and the Wright-Fisher process^15^ have become popular models to describe how phenotypes change over time by evolution. Both processes have their roots in population genetics. Only recently, they were introduced to evolutionary game dynamics in finite populations^6,16,17^. In each time step of the *Moran process*, an individual is selected proportional to its fitness and produces an identical offspring. Subsequently, another randomly chosen individual is removed from the population. In the *Wright-Fisher process*, all individuals produce a large number of identical offspring based on their fitness. Then, *N* of the offspring individuals are selected randomly to become the next generation population. We are focusing on computations for the Moran process here. Other processes are considered as possible extensions in the discussion. We also focus on discrete-time processes. Continuous-time processes require different simulation techniques (e.g. based on the Gillespie algorithm^18^) that are beyond our scope, see^19^ for a systematic comparison of these processes.

In evolutionary game dynamics, interactions between types are defined by a *payoff matrix*. We consider a population of *N* individuals with two types or strategies, *A* and *B*. The payoff matrix is given by $$(\begin{array}{cc}a & b\\ c & d\end{array})$$ and describes the payoff that each type gets from interaction with its own and the other type respectively. If two *A*’s interact, they both get payoff *a*. If an *A* meets a *B*, *A* gets *b*, whereas *B* gets *c*. If two *B*’s interact, they both get payoff *d*.

A key quantity is the *fitness* which measures how successfully a type (e.g. phenotype/strategy) reproduces. In the context of evolutionary dynamics in finite populations, it has a direct interpretation in terms of relative birth and death rates^20^. In the Moran process, the selection mechanism can be thought of as a roulette wheel, in which every field represents one individual and the higher its fitness, the larger the field on the wheel^21^. In classic population genetics, the fitness is usually only dependent on the focal individuals’ type. In evolutionary game theory, however, it is often partitioned into two parts *f* = *f*_0_ + *βπ*: a constant background fitness which is independent of other individuals, e.g. *f*_0_ = 1, and a payoff which is dependent on others, *π*.

The *selection intensity β* represents how strongly fitness depends on the game. For strong selection, $\beta N\gg 1$, the evolutionary game dominates the dynamics. In a weak selection regime on the other hand, $\beta N\ll 1$, the dynamics are mostly stochastic^22,23^.

In general, any *payoff-to-fitness mapping f* is assumed to avoid negative fitness in games where payoffs can be negative. Additionally it should be an increasing function of the payoff ^23,24^. For the linear payoff-to-fitness mapping, *β* has to be bound in order to keep the fitness positive. By using an exponential payoff-to-fitness mapping, *f* = exp(*βπ*)^23,25^, this bound on *β* is not necessary. We will focus on this mapping here. It is standard in a range of applications^26–30^ and analytically convenient by allowing to replace a product by a sum, namely the product of transition ratios that appears for calculating fixation probability. At the same time, the exponential mapping approximates the results of the simple linear payoff-to-fitness mapping when *β* is sufficiently small.

The Moran process and the Wright-Fisher process share a lot of similarities: (a) they are both represented by absorbing Markov chains, (b) they keep the population size constant, (c) they have the same absorbing states where every individual has the same strategy (either all *A* or all *B*). In particular, because of (c), it is interesting to ask for the probability that each of these absorbing states is reached, given a certain initial condition. We focus on the probability that a single mutant takes over the population of wild-type individuals, i.e. the *fixation probability* in a population of two types.

Besides the fixation probability, the time it takes a mutant to take over a population is of interest. The average *unconditional fixation time* is defined as the number of time steps it takes starting from one mutant until extinction or fixation of the mutants. As the population is assumed to be finite, the process hits one of the absorbing boundaries after finite time with probability 1. Another interesting quantity of the process is the *conditional fixation time*. It is given by the time it takes one mutant to take over the population, given that it does succeed. For simulating the conditional fixation time, this means only keeping track of the time steps of realisations where the mutant takes over and discarding the runs where the mutants go extinct.

When we introduce mutations, the homogeneous population states are not absorbing anymore. In that case, we are interested in the *stationary distribution* of the process. For every state of the population, the stationary probability distribution gives the probability that the process is at that state in the long run.

Another process we will mention is the *pairwise comparison process* with the Fermi function^23,31,32^. Instead of letting an individual reproduce based on fitness, a pair of a focal individual and a role model are randomly chosen in each time step. The focal individual evaluates its payoff difference using an imitation function. This determines the probability that the focal individual adopts the strategy of the role model. As this process is a simple birth-death process, it shares the same complexity as the Moran process for computing the above mentioned quantities.

It is important to note that there are also alternative approaches to evolutionary dynamics in finite populations, other than the ones we discuss here. In particular, stochastic differential equations are useful to derive mean-field predictions from individual based models if the population size is finite, but large^33–35^. These alternative methods may be particularly useful when the population size is large enough that it renders the methods we discuss unfeasible due to computational complexity, or when specific features such as spatial structure combine with large population sizes^36–38^. Note, however, that as the population size becomes very large the stochastic effects we are concerned with become less important.

**Figure 1:**
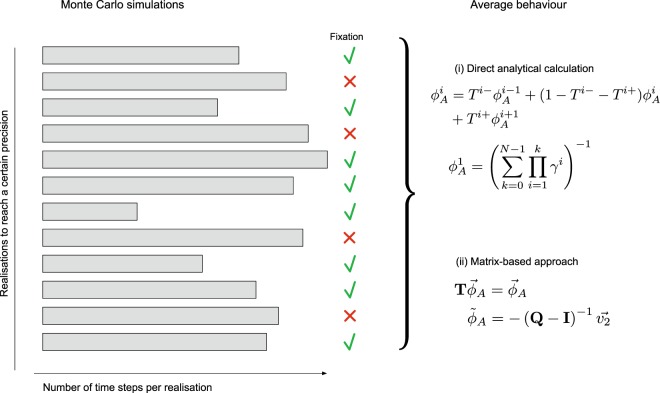
Schematic picture of the wall time for simulating fixation in an evolutionary process. The wall time comprises a number of realisations, necessary to reach a certain precision; as well as the number of time steps each realisation takes until fixation or extinction. The length of each fixation event depends on the underlying game and the selection intensity. When simulating the conditional fixation time, the realisations that lead to extinction are discarded. How many steps are discarded depends on the fixation probability. All realisations are used when computing the unconditional fixation time.

Thinking about an evolutionary process in a computational way can deliver insights into the details of the process. This becomes apparent, for example, when thinking about the wall time required to simulate a process in order to reach a target precision. The *wall time* is the actual time that elapses between the start and the end of a program. When simulating an evolutionary process, the wall time is composed of the number of realisations and the time each realisation takes before the process hits an absorbing state, see the conceptual Fig. 1. A very high fixation probability requires few realisations (see Section *When to stop the simulation?*). However, there are situations where high fixation probability occurs together with high fixation time, which entails that it takes longer to simulate each realisation. Understanding these tradeoffs between few realisations necessary to simulate a high fixation probability occurring together with a high fixation time that might need a high number of time steps can be insightful and useful.

## Methods

We discuss three methods to calculate the fixation probabilities, the fixation times, and the stationary distribution. These three main methods, which also define the underlying structure in this paper, are:

(i) a direct analytical solution
(ii) a numerical approach based on the transition matrix of the associated Markov chains
(iii) Monte Carlo simulations.

As our results are intimately connected to the details of implementation, further details are given in the results sections.

Analytical solutions are usually the most elegant, but they are often convoluted in practice and only limiting cases, for example arising from small intensity of selection *β*, can be interpreted easily. The naive implementations of the full analytical results are sometimes inefficient and can be computationally more expensive than smart simulations.

Alternatively, the numerical approach based on the transition matrix of the Markov chain can be useful and can feel natural when thinking about the process in terms of transition probabilities. However, as the transition matrix size grows quadratically with population size, this computational approach becomes unfeasible for large populations in terms of memory^39^ and even much faster for graph structured populations, where the transition matrix can be of size 2^*N*^ × 2^*N*^^40,41^. Making use of sparse solvers for banded matrices, however, leads to linear convergence of the computation time with population size in the case without population structure.

To discuss these methods, we focus mostly on the Moran process, mentioning the alternative Wright-Fisher process occasionally as an extension.

The source code and demo notebooks can be downloaded from http://bit.ly/finite_computation_ed.

## Results

### Direct analytical calculation

#### Fixation probability

The direct analytical calculation is based on the solution of a recursive equation to receive the desired quantities. Let us show this by using the Moran process with two strategies, *A* and *B*, as an example. The payoff matrix is given by $$(\begin{array}{cc}a & b\\ c & d\end{array})$$. If two *A*’s interact, they both get payoff *a*. If an *A* meets a *B*, *A* gets *b*, whereas *B* gets *c*. If two *B*’s interact, they both get payoff *d*. Let *i* be the number of strategy *A* individuals in a population of size *N*. For the Moran process, in every time step, *i* can only increase or decrease by one or stay the same. Let us denote *T*^*i*+^ as the probability that *i* increases by one and *T*^*i*−^ as the probability that *i* decreases by one.

Here, we are interested in the probability ${\varphi }_{A}^{i}$ that the population reaches fixation of *A* when initially there are *i* strategy *A* individuals in the population. Without mutations, the boundary conditions are given by ${\varphi }_{A}^{0}=0$ and ${\varphi }_{A}^{N}=1$: If there are only *B*-strategists, the probability that the *A*-strategists take over is zero. Similarly, if the population consists of only type *A*, the fixation probability of them is one. Based on the forward Kolmogorov equation^8,21^, we have

1 $${\varphi }_{A}^{i}={T}^{i-}{\varphi }_{A}^{i-1}+\mathrm{(1}-{T}^{i-}-{T}^{i+}){\varphi }_{A}^{i}+{T}^{i+}{\varphi }_{A}^{i+1}\mathrm{.}$$

Solving the recursion yields the fixation probability of a single type *A* individual invading a population^6,21,42^

2 $${\varphi }_{A}^{1}={(\sum _{k=0}^{N-1}\prod _{i=1}^{k}{\gamma }^{i})}^{-1},$$

where *γ*^*i*^ = *T*^*i*−^/*T*^*i*+^ and where the empty product is defined as 1.

For the Moran process with a payoff-to-fitness mapping $f={e}^{\beta {\pi }^{i}}$, let us denote ${\pi }_{A}^{i}$ and ${\pi }_{B}^{i}$ as the payoff for a single strategy *A* and *B* individual when there are *i* individuals playing strategy *A*. These payoffs determine the transition probabilities via the fitness^6,25^,

3 $$\begin{array}{rcl}{T}^{i+} & = & \frac{i{e}^{\beta {\pi }_{A}^{i}}}{i{e}^{\beta {\pi }_{A}^{i}}+(N-i){e}^{\beta {\pi }_{B}^{i}}}\frac{N-i}{N},\\ {T}^{i-} & = & \frac{(N-i){e}^{\beta {\pi }_{B}^{i}}}{i{e}^{\beta {\pi }_{A}^{i}}+(N-i){e}^{\beta {\pi }_{B}^{i}}}\frac{i}{N}.\end{array}$$

This leads to ${\gamma }^{i}=\exp [\beta ({\pi }_{B}^{i}-{\pi }_{A}^{i})]$.

It is of common interest to ask for which selection intensity the fixation probability is greater than that of the neutral case, where we have ${\varphi }_{A}^{1}(\beta =\mathrm{0)}=1/N$. Theoretical insights are difficult to obtain based on equation (2). This is because the equation ${\varphi }_{A}^{1}(\beta )=1/N$ is typically transcendental for non-linear payoff-to-fitness mapping. Even for the linear payoff-to-fitness mapping, the equation contains a polynomial of order *N* in the denominator. Weak selection, i.e. $\beta \ll 1$, can provide substantial further insight^6,24,28,43,44^ because it usually simplifies analytical calculations.

The fixation probability can then be approximated by Taylor expansion

4 $${\varphi }_{A}^{1}(\beta )\approx \frac{1}{N}+\mathop{\underbrace{f^{\prime} \mathrm{(0)}}}\limits_{\ge 0}\beta \mathop{\underbrace{\sum _{k=0}^{N-1}\,\sum _{i=1}^{k}\,({\pi }_{A}^{i}-{\pi }_{B}^{i})}}\limits_{D}.$$

When *D* > 0, ${\varphi }_{A}^{1}(\beta ) > 1/N$, such that selection favors the invasion of strategy *A* under weak selection. An alternative approximation is to replace the sum and the product in equation (2) by integrals, but the resulting expression is still difficult to interpret^22^. However, if we are interested in exact results for general selection intensities and population sizes, we need to resort to numerical techniques.

Having the formula at hand, we transform equation (2) into **Algorithm 1** to compute this quantity.

**Algorithm 1:**
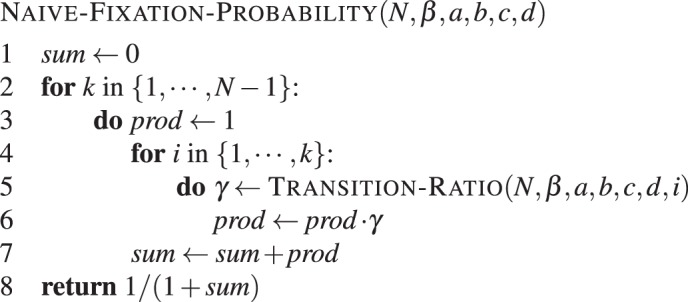
Direct fixation probability (naive version). Fixation of type *A* in a population of *N* − 1 individuals of type *B*, with intensity of selection *β* and game given by *a*, *b*, *c* and *d*.

Here, the function TRANSITION-RATIO(*N*, *β*, *a*, *b*, *c*, *d*, *k*) implements the formula ${e}^{\beta ({\pi }_{B}-{\pi }_{A})}$ with ${\pi }_{A}=\frac{a(k-1)+b(N-k)}{N-1}$ and ${\pi }_{B}=\frac{ck+d(N-k-1)}{N-1}$, the payoffs of type A and B, respectively. This naive implementation results in two nested loops. Note that we can store the product (line 6), such that we can reduce to a single loop. A pseudo-code that avoids a second loop is given by **Algorithm 2**.

**Algorithm 2:**
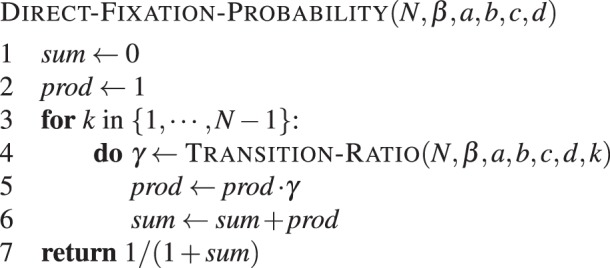
Direct fixation probability. Fixation of type *A* in a population of *N* − 1 individuals of type *B*, with intensity of selection *β* and game given by *a*, *b*, *c* and *d*.

Computing the ratio of transition probabilities in line 4 of DIRECT-FIXATION-PROBABILITY() does not depend on *N*, thus we obtain a scaling in ${\mathscr{O}}\mathrm{(1)}$.

The loop is entered *N* − 1 times. Thus, the time-complexity of the whole computation is of order ${\mathscr{O}}(N)$.

Note that the naive implementation in **Algorithm 1** with two nested loops, results in *N*(*N* − 1)/2 computations of the transition ratio *γ*^*i*^, providing a less efficient computation of quadratic order.

The above computation works for arbitrary intensity of selection *β*. Weak selection is often used as it leads to closed formulas as shown in equation (4), but if numerics are required, the term *D* in equation (4) will still lead to a linear time complexity computation. The weak selection approximation can be theoretically insightful, in particular when the sums can be solved analytically (such as for two-player matrix games or multiplayer games^45^), but it is in general not computationally more efficient than the case of general *β*.

#### Unconditional fixation time

We can also use a direct analytical computation for computing the average number of steps required for fixation. The expected unconditional fixation time *τ*^*i*^, starting from *i* individuals of type *A*, can be recursively calculated from^8,21^

5 $${\tau }^{i}=1+{T}^{i-}{\tau }^{i-1}+(1-{T}^{i-}-{T}^{i+}){\tau }^{i}+{T}^{i+}{\tau }^{i+1},$$

where the transition probabilities *T*^*i*−^ and *T*^*i*+^ are given by equation (3). The boundary conditions are *τ*^0^ = *τ*^*N*^ = 0. Solving the recursion, one obtains the expected unconditional fixation time *τ*^1^, starting from a single individual^21^

6 $${\tau }^{1}={\varphi }_{A}^{1}\,\sum _{k=1}^{N-1}\,\sum _{l=1}^{k}\,\frac{1}{{T}^{l+}}\prod _{m=l+1}^{k}\,\frac{{T}^{m-}}{{T}^{m+}},$$

where ${\varphi }_{A}^{1}$ is given by (2). Again one can obtain additional insights from a weak selection approximation of this quantity^24,46–48^.

For computational reasons (explained in Supplementary Method *Calculating the unconditional fixation time*), we rewrite the above equation as

7 $${\tau }^{1}={\varphi }_{A}^{1}\,\sum _{l=1}^{N-1}\,\frac{{R}^{l}}{{T}^{(N-l)+}}.$$

where *R*^*l*^ can be calculated recursively from

8 $${R}^{l+1}=1+{\gamma }^{N-l}{R}^{l},$$

with *R*^1^ = 1 (see Supplementary Method *Calculating the unconditional fixation time*). This simplification holds for general selection intensity *β*.

Using equations (7) and (8), the computation is simplified and can be executed as presented in **Algorithm 3**.

**Algorithm 3:**
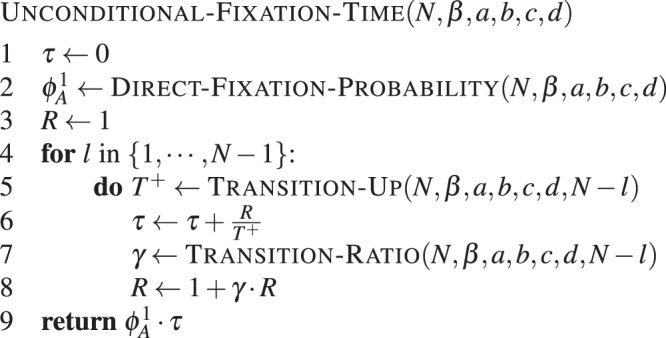
Direct unconditional fixation time. Unconditional fixation time of type *A* in a population of *N* − 1 individuals of type *B*, with intensity of selection *β* and game given by *a*, *b*, *c* and *d*.

which uses the function TRANSITION-UP(*N*, *β*, *a*, *b*, *c*, *d*, *l*), implementing the probability that the number of *A*-strategists increases by one in one time-step. This is given by *T*^+^ as follows:

$${T}^{+}=\frac{l\,{f}_{A}}{l\,{f}_{A}+(N-l){f}_{B}}\frac{N-l}{N}$$

where fitnesses are ${f}_{A}={e}^{\beta {\pi }_{A}}$ and ${f}_{B}={e}^{\beta {\pi }_{B}}$, with payoffs

$${\pi }_{A}=\frac{a(l-1)+b(N-l)}{N-1}$$ and $${\pi }_{B}=\frac{cl+d(N-l-1)}{N-1}$$.

The modules TRANSITION-RATIO() and DIRECT-FIXATION-PROBABILITY() are defined as in **Algorithm 2**. The complexity of calculating the fixation probability in line 3 of UNCONDITIONAL-FIXATION-TIME() is of order *N*. The computation time of the payoff ratio *γ* does not depend on *N*, so it has constant time complexity. The summation loop is entered *N* − 1 times. Therefore, the time-complexity of the whole calculation is of the order ${\mathscr{O}}(N)$.

#### Conditional fixation time

A Master equation for the expected conditional fixation time ${\tau }_{A}^{i}$, starting in state *i* and fixating in state *N*, is given by^8,21,49^

9 $${\varphi }_{A}^{i}{\tau }_{A}^{i}=(1-{T}^{i+}-{T}^{i-}){\varphi }_{A}^{i}{\tau }_{A}^{i}+{T}^{i-}{\varphi }_{A}^{i-1}({\tau }_{A}^{i-1}+1)+{T}^{i+}{\varphi }_{A}^{i+1}({\tau }_{A}^{i+1}+1),$$

where ${\tau }_{A}^{0}=0$ and ${\tau }_{A}^{N}=0$. Solving the recursion yields^8,21,42,50^

10 $${\tau }_{A}^{1}=\sum _{k=1}^{N-1}\,\sum _{l=1}^{k}\,\frac{{\varphi }_{A}^{l}}{{T}^{l+}}\,\prod _{m=l+1}^{k}\,\frac{{T}^{m-}}{{T}^{m+}}.$$

The above equation can be rewritten as

11 $${\tau }_{A}^{1}=\sum _{l=1}^{N-1}\,\frac{{\psi }_{A}^{l}}{{T}^{(N-l)+}}{R}^{l}$$

for general *β*, where the following recursions hold

12 $$\begin{array}{cc}{R}^{l+1}=1+{\gamma }^{N-l}{R}^{l}, & {\rm{w}}{\rm{i}}{\rm{t}}{\rm{h}}\,{R}^{1}=1,\\ {\psi }_{A}^{h}={\psi }_{A}^{h-1}-{\varphi }_{A}^{1}(\prod _{m=1}^{N-h}{\gamma }^{m}), & {\rm{w}}{\rm{i}}{\rm{t}}{\rm{h}}\,{\psi }_{A}^{1}={\varphi }_{A}^{N-1}\end{array}$$

see Supplementary Method *Calculating the conditional fixation time*. This is expressed in **Algorithm 4**.

The modules DIRECT-FIXATION-PROBABILITY(), TRANSITION-RATIO() and TRANSITION-UP() are defined as in **Algorithm 1** and in **Algorithm 3**.

**Algorithm 4:**
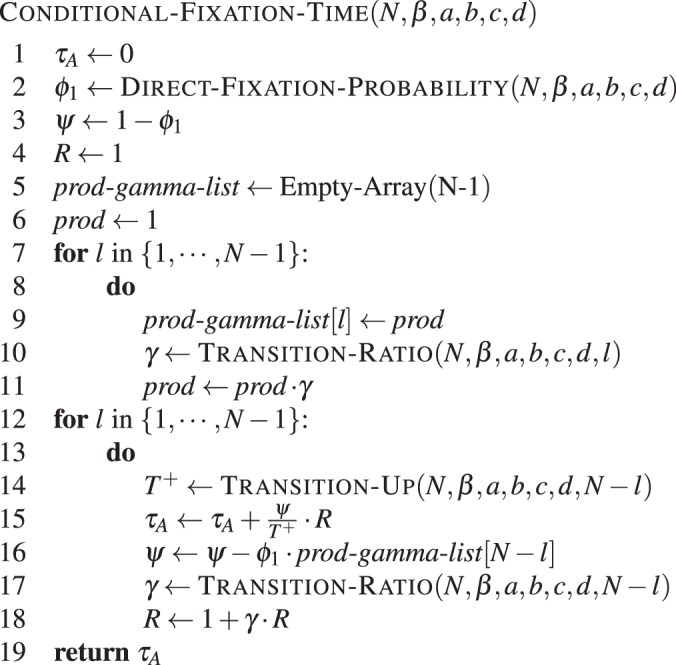
Direct conditional fixation time. Conditional fixation time of type *A* in a population of *N* − 1 individuals of type *B*, with intensity of selection *β* and game given by *a*, *b*, *c* and *d*.

The complexity of calculating the fixation probability is of order ${\mathscr{O}}(N)$ (line 2). Again, the computation time of the transition ratio does not depend on *N*. We have to use memorisation to store the product of transition ratios *γ*. Therefore we have two loops that are entered *N* − 1 times. The time-complexity of the whole calculation is still ${\mathscr{O}}(N)$. Note that it takes much longer if we calculate the conditional fixation time directly based on equation (10), where one would naively come up with an algorithm that implements each sum and product separately, leading to ${\mathscr{O}}({N}^{3})$.

An alternative approach to calculate fixation times can be implemented via the sojourn times^15,51–53^. The average *sojourn time* in a transient state *i* ∈ {1, …, *N* − 1} gives the average number of time steps the process spends in that state before absorption. Summing up the sojourn times of all transient states gives the average fixation time. While this calculation can lead to additional insight^51^, that approach does not lead to a further reduction in computation time.

#### Stationary distribution

So far, we have considered the fixation of either of the two types. The process will eventually hit one of the absorbing boundaries with probability 1 in the absence of mutations. In the presence of mutations, however, the types can no longer fixate in the population. Instead of the fixation probability and time, we then study the stationary probability distribution. The stationary probability distribution gives the fraction of time the process spends in each state in the long run^39^. Mutations are implemented as in^54^ with mutation rate *μ*. The transition probabilities including mutation are^54^

13 $${T}^{k+}=\frac{k\,{f}_{A}}{k\,{f}_{A}+(N-k){f}_{B}}\frac{(N-k)}{N}(1-\mu )+\frac{(N-k){f}_{B}}{k\,{f}_{A}+(N-k){f}_{B}}\frac{(N-k)}{N}\mu $$

14 $${T}^{k-}=\frac{(N-k){f}_{B}}{k\,{f}_{A}+(N-k){f}_{B}}\frac{k}{N}(1-\mu )+\frac{k\,{f}_{A}}{k\,{f}_{A}+(N-k){f}_{B}}\frac{k}{N}\mu $$

where the first part in *T*^*k*+^ (*T*^*k*−^) corresponds to choosing a mutant (wild-type) for birth, a wild-type (mutant) for death and no mutation happening. The second part describes the probability of choosing the same types for birth and death, but a mutation happening.

For general mutation rate *μ* and selection strength *β*, the stationary probability distribution *p*^*k*^ can be calculated from detailed balance^55^. It is given by^54,56,57^

15 $${p}^{k}={p}^{k-1}\frac{{T}^{(k-1)+}}{{T}^{k-}}={p}^{0}\prod _{i=0}^{k-1}\,\frac{{T}^{i+}}{{T}^{(i+1)-}},$$

where *p*^0^ can be obtained from normalisation, ${\sum }_{k=0}^{N}\,{p}^{k}=1$.

The pseudo-code for computing the stationary distribution is given by **Algorithm 5**, which uses the functions TRANSITION-UP(*N*, *β*, *a*, *b*, *c*, *d*, *k*) and TRANSITION-DOWN(*N*, *β*, *a*, *b*, *c*, *d*, *k*), implementing the probability that the number of *A*-strategists increases or decreases by one, respectively. These are given by equation 13 and 14.

**Algorithm 5:**
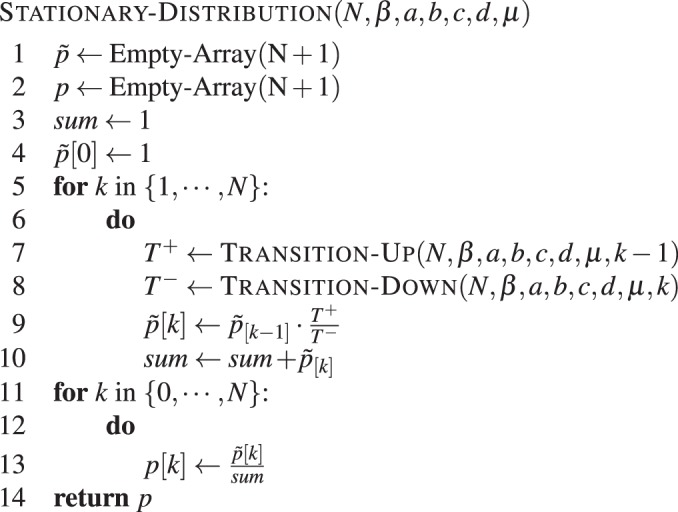
Direct stationary distribution. Stationary distribution for two types in a population of size *N*, with intensity of selection *β*, mutation probability *μ*, and game given by *a*, *b*, *c* and *d*.

The computation of the stationary distribution with this algorithm scales in ${\mathscr{O}}(N)$.

#### Limitations and scalability

The fixation probability in equation (2) is valid for any one-dimensional birth-death process. Thus, in particular it also applies to imitation processes^23,24^ as well as to general multiplayer games^45,58^, where the payoff depends on the state of the population in a polynomial way. The Moran process on a cycle-graph also reduces to a one-dimensional birth-death process and thus falls into that category^53,59^. It is important to see that these applications do not change the order of the time-complexity, and only affect the computational time by constant factors that do not depend on population size.

This method does not work for the Wright-Fisher process, because the Wright-Fisher process is not a birth-death process. In one time step the number of *A* individuals can change by more than one. However, the diffusion approximation provides a very powerful way to approximate the fixation probability accurately^15,17,60^. This typically involves two nested integrals, which implies the same computational complexity as our nested double sums, assuming that the discretisation of the integrals uses 1/*N* as a step size.

Calculating equation (2) can lead to computational inaccuracies in some specific cases. Summing up numbers in floating point representation will carry truncation errors that are no longer negligible if summing up many numbers. Thus, if the population size is large, this issue needs to be addressed. A number of algorithms can be used to alleviate the problem. A discussion of those can be found in^61,62^. Issues may also arise when *γ*^*i*^ values are either too small or too large (leading to numerical underflow/overflow). These often appear when computing fixation probabilities for strong selection. For example, for payoff matrix $$(\begin{array}{cc}1 & 2\\ 3 & 4\end{array})$$, with population size *N* = 20, *γ*^10^(*β*) = exp[*β*(*π*_*B*_ − *π*_*A*_)] ≈ 10^84^ for strong selection, *β* = 100.

**Figure 2:**
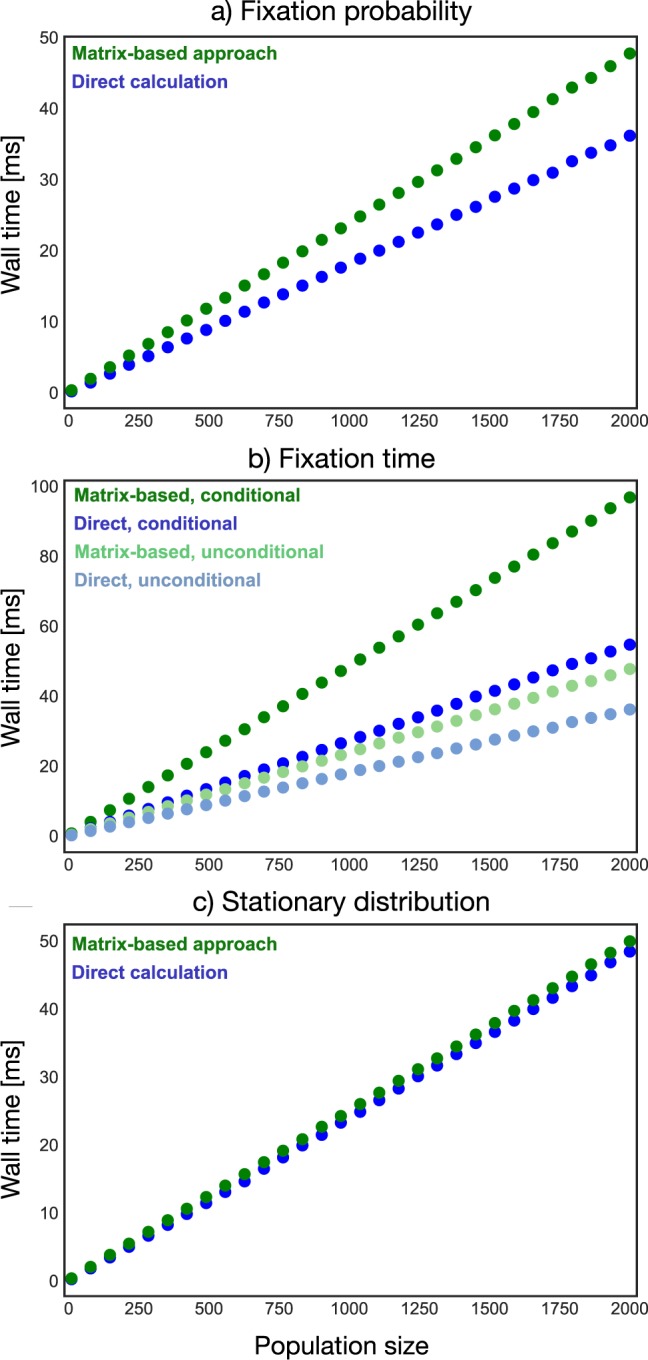
Empirical measure of the wall time for computing the (**a**) fixation probability, (**b**) average conditional and unconditional fixation time and (**c**) stationary distribution. All wall times grow linearly with population size. The wall time for all these computations is independent of the game and selection intensity. The parameters used for the computations were selection intensity *β* = 1 and payoffs *a* = 2, *b* = 5, *c* = 1, *d* = 3. For panel (c) a mutation rate of *μ* = 0.1 was used.

In summary, a naive implementation of the direct calculation will lead to quadratic complexity in fixation probability. This directly affects computations of fixation time that rely on the fixation probability. But all quantities of interest here can be computed in linear time with the appropriate implementation, c.f. Fig. 2.

### Numerical matrix-based approach

In this section, we will again make use of recursions to estimate the same three important quantities in evolutionary theory: fixation probability, fixation times and stationary distribution. For all the three quantities, the main idea is the same, i.e., to make use of the Kolmogorov backward equation to get a linear difference equation^39^. Yet for different quantities, the recursive equation comes with different boundary conditions. Consequently, a standard method based on matrix algebra would facilitate obtaining the quantities analytically.

#### Fixation probability

For the Moran process of two types in a well-mixed population, the state space is determined by *i*, the number of individuals playing strategy *A*, *i* ∈ {0, 1, …, *N*}. The fixation probability can be recursively calculated from the Master equation given by equation (1).

In this process, it is only possible to increase or decrease *i* by one, so the transition matrix is tridiagonal and given by the matrix ***T***^(*N* + 1) × (*N* + 1)^ with elements

$$(\begin{array}{cccccccccc}{T}^{0\circ } & {T}^{0+} & 0 & \cdots  & \cdots  & \cdots  & \cdots  & \cdots  & \cdots  & 0\\ {T}^{1-} & {T}^{1\circ } & {T}^{1+} & 0 & \cdots  & \cdots  & \cdots  & \cdots  & \cdots  & 0\\ 0 & {T}^{2-} & {T}^{2\circ } & {T}^{2+} & 0 & \cdots  & \cdots  & \cdots  & \cdots  & 0\\ \vdots  & \ddots  & \ddots  & \ddots  & \ddots  & \ddots  &  &  &  & \vdots \\ \vdots  &  & \ddots  & \ddots  & \ddots  & \ddots  & \ddots  &  &  & \vdots \\ \vdots  &  &  & \ddots  & \ddots  & \ddots  & \ddots  & \ddots  &  & \vdots \\ \vdots  &  &  &  & \ddots  & \ddots  & \ddots  & \ddots  & \ddots  & \vdots \\ \vdots  &  &  &  &  & 0 & {T}^{(N-2)-} & {T}^{(N-2)\circ } & {T}^{(N-2)+} & 0\\ 0 & \cdots  & \cdots  & \cdots  & \cdots  & \cdots  & 0 & {T}^{(N-1)-} & {T}^{(N-1)\circ } & {T}^{(N-1)+}\\ 0 & \cdots  & \cdots  & \cdots  & \cdots  & \cdots  & \cdots  & 0 & {T}^{N-} & {T}^{N\circ }\end{array})$$

where ° denotes the probability to stay in a state. Note that the states 0 and *N* are absorbing and therefore *T* ^0°^ = *T*^*N*°^ = 1 and *T* ^0+^ = *T*^*N*−^ = 0.

Let us write this as a block matrix, where ***Q***^(*N* − 1) × (*N* − 1)^ is the transition matrix between transient states^39,41^

16 $$\begin{array}{ccc}{\bf{T}} & = & (\begin{array}{lll}1 & {\overrightarrow{0}}^{T} & 0\\ {\overrightarrow{v}}_{1} & {\bf{Q}} & {\overrightarrow{v}}_{2}\\ 0 & {\overrightarrow{0}}^{T} & 1\end{array})\end{array}.$$

We can now write the Master equation (1) for the fixation probabilities as an eigenvector problem,

17 $${\bf{T}}{\overrightarrow{\varphi }}_{A}={\overrightarrow{\varphi }}_{A},$$

where ${\overrightarrow{\varphi }}_{A}={({\varphi }_{A}^{0},{\varphi }_{A}^{1},\ldots ,{\varphi }_{A}^{N})}^{T}$. If we use the block formulation of equation (16), then equation (17) is equivalent to

18a $$1{\varphi }_{A}^{0}+{\overrightarrow{0}}^{T}{\tilde{\varphi }}_{A}+0{\varphi }_{A}^{N}={\varphi }_{A}^{0}=\mathrm{0,}$$

18b $${\overrightarrow{v}}_{1}{\varphi }_{A}^{0}+{\bf{Q}}{\tilde{\varphi }}_{A}+{\overrightarrow{v}}_{2}{\varphi }_{A}^{N}={\tilde{\varphi }}_{A}$$

18c $$0{\varphi }_{A}^{0}+{\overrightarrow{0}}^{T}{\tilde{\varphi }}_{A}+1{\varphi }_{A}^{N}={\varphi }_{A}^{N}=1.$$

where ${\tilde{\varphi }}_{A}={({\varphi }_{A}^{1},{\varphi }_{A}^{2},\ldots ,{\varphi }_{A}^{N-1})}^{T}$. It is noteworthy that equations (18a) and (18c) always hold. Thus, we need only solve equation (18b). Using ${\varphi }_{A}^{0}=0$ and ${\varphi }_{A}^{N}=1$, equation (18b) can be written as

19 $$({\bf{Q}}-{\bf{I}}){\tilde{\varphi }}_{A}=-\,{\overrightarrow{v}}_{2},$$

which is explicitly given by

20 $$\begin{array}{lllll}(\begin{array}{lllllll}{T}^{1\circ }-1 & {T}^{1+} & 0 & \cdots  & \cdots  & \cdots  & 0\\ {T}^{2-} & {T}^{2\circ }-1 & {T}^{2+} & \cdots  & \cdots  & \cdots  & 0\\ \vdots  &  &  & \ddots  &  &  & \vdots \\ 0 & \cdots  & \cdots  & \cdots  & {T}^{(N-\mathrm{2)}-} & {T}^{(N-\mathrm{2)}\circ }-1 & {T}^{(N-\mathrm{2)}+}\\ 0 & \cdots  & \cdots  & \cdots  & 0 & {T}^{(N-\mathrm{1)}-} & {T}^{(N-\mathrm{1)}\circ }-1\end{array}) & \cdot  & (\begin{array}{c}{\varphi }_{A}^{1}\\ {\varphi }_{A}^{2}\\ \vdots \\ {\varphi }_{A}^{N-2}\\ {\varphi }_{A}^{N-1}\end{array}) & = & -(\begin{array}{c}0\\ 0\\ \vdots \\ 0\\ {T}^{(N-\mathrm{1)}+}\end{array})\end{array}$$

Now this matrix system has to be solved for ${\tilde{\varphi }}_{A}$. Note that for none of the steps it is necessary to have a tridiagonal matrix.

We can now formulate an algorithm to compute fixation probabilities. We first build a transition matrix, omitting the absorbing states from the matrix, such that we have **Q** instead of **T**. Then the vector *v*_2_ is created as a vector of zeros and its last element is set to the transition probability *T*^(*N* − 1)+^. Third, we subtract the identity matrix from **Q**. The last part of the algorithm is to compute the solution to a standard system of linear equations. This procedure is given by **Algorithm 6**.

**Algorithm 6:**
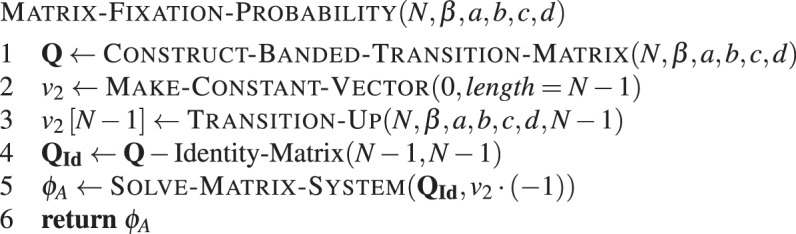
Matrix-based fixation probability. Fixation of type *A* in a population of *N* − 1 individuals of type *B*, with intensity of selection *β* and game given by *a*, *b*, *c* and *d*.

For general transition matrices, the time complexity of line 1 would be quadratic. But noting that the transition matrix is tridiagonal, we can perform this step in linear time when using an appropriate representation for the banded matrix. The first step is thus, ${\mathscr{O}}(N)$.

The vector construction can be performed in linear time and the same holds for subtracting 1 from the diagonal of a banded matrix. Since the resulting matrix is also tridiagonal, the time complexity of solving the linear system is ${\mathscr{O}}(N)$^63^.

In summary, a naive implementation will lead to quadratic time complexity. But profiting from the tridiagonal nature of the system when dealing with birth-death processes, we can achieve the same results in linear time.

In the Wright-Fisher process we can use a similar procedure, relying on a much denser transition matrix. The probability of transitions between states have been discussed by^17^. In this case, however, the resulting matrix is not tridiagonal, and therefore complexity is at least ${\mathscr{O}}({N}^{2})$, resulting both from the construction of the matrix and the solution of the linear system, which typically scales with ${\mathscr{O}}({N}^{3})$.

#### Unconditional fixation time

Reformulating equation (5), we can write the calculation of the unconditional fixation times as an eigenvector problem^41^

21 $${\mathscr{T}}\,\overrightarrow{\tau }=\overrightarrow{\tau },$$

where $\overrightarrow{\tau }={(1,{\tau }^{0},{\tau }^{1},\ldots ,{\tau }^{N})}^{T}$ and the modified transition matrix is given by

22 $${\mathscr{T}}=(\begin{array}{cc}1 & {\overrightarrow{0}}^{T}\\ {\overrightarrow{v}}_{0} & {\bf{T}}\end{array})=(\begin{array}{cccc}1 & 0 & {\overrightarrow{0}}^{T} & 0\\ 0 & 1 & {\overrightarrow{0}}^{T} & 0\\ \overrightarrow{1} & {\overrightarrow{v}}_{1} & {\bf{Q}} & {\overrightarrow{v}}_{2}\\ 0 & 0 & {\overrightarrow{0}}^{T} & 1\end{array}),$$

where ${\overrightarrow{v}}_{0}={(0,1,\ldots ,1,0)}^{T}$ and $\overrightarrow{1}={(1,1,\ldots ,1)}^{T}$. Expressing equation (5) in block matrix notation, yields

23a $$1=1$$

23b $$0+1{\tau }^{0}+{\overrightarrow{0}}^{T}\tilde{\tau }+0{\tau }^{N}={\tau }^{0}=0,$$

23c $$\overrightarrow{1}+{\overrightarrow{v}}_{1}{\tau }^{0}+{\bf{Q}}\tilde{\tau }+{\overrightarrow{v}}_{2}{\tau }^{N}=\tilde{\tau },$$

23d $$0+0{\tau }^{0}+{\overrightarrow{0}}^{T}\tilde{\tau }+1{\tau }^{N}={\tau }^{N}=0,$$

where $\tilde{\tau }={({\tau }^{1},\ldots ,{\tau }^{N-1})}^{T}$. Equations (23a), (23b) and (23d) always hold. Therefore, we only have to solve equation (23c). Subtracting $\tilde{\tau }$ and $\overrightarrow{1}$ on both sides, we obtain a matrix equation

24 $$({\bf{Q}}-{\bf{I}})\tilde{\tau }=-\,\,\overrightarrow{1}\mathrm{.}$$

Now this matrix system has to be solved for $\tilde{\tau }$.

Similar to **Algorithm 6**, we have to construct the transition matrix first, then create a vector of ones and solve the matrix system.

**Algorithm 7:**
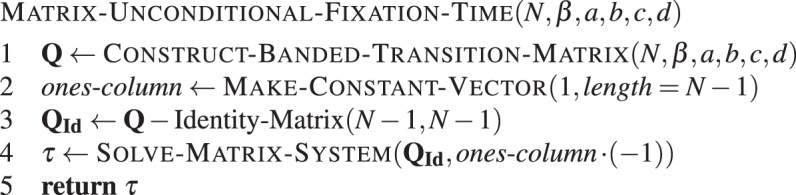
Matrix-based unconditional fixation time. Unconditional fixation time of type *A* in a population of *N* − 1 individuals of type *B*, with intensity of selection *β* and game given by *a*, *b*, *c* and *d*.

As the transition matrix is tridiagonal, i.e. a banded matrix with band 1, all operations run in ${\mathscr{O}}(N)$. A similar approach can be applied to a Wright-Fisher process, where a non-banded matrix will result in cubic wall time at most.

#### Conditional fixation time

Before solving the recursive equation to compute the conditional fixation time, we have to modify the transition probabilities by weighting them by a ratio of fixation probabilities^41,52,64^. The conditional transition probabilities now read

25 $${T}_{A}^{i\,+}=\frac{{\varphi }_{A}^{i+1}}{{\varphi }_{A}^{i}}{T}^{i+}$$

26 $${T}_{A}^{i\,-}=\frac{{\varphi }_{A}^{i-1}}{{\varphi }_{A}^{i}}{T}^{i-}.$$

Calculating these weighted transition probabilities first, one can compute the conditional fixation times by following the same approach as for the unconditional fixation times above. Note that this is computationally more expensive, as the linear system for the fixation probabilities has to be solved first.

For the conditional fixation time, the algorithm is similar to **Algorithm 7** above, except that the transition matrix has to be modified first. For this, we use the fixation probabilities computed with **Algorithm 6**.

**Algorithm 8:**
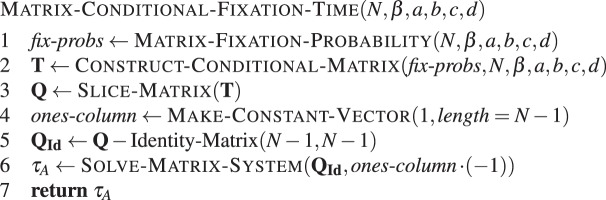
Matrix-based conditional fixation time. Conditional fixation time of type *A* in a population of *N* − 1 individuals of type *B*, with intensity of selection *β* and game given by *a*, *b*, *c* and *d*.

As discussed above, the algorithm MATRIX-FIXATION-PROBABILITY() scales in ${\mathscr{O}}(N)$. Weighting the transition probabilities by the fixation probabilities scales linear in time, because the transition matrix is tridiagonal. The same holds for solving the system, therefore the whole algorithm runs in ${\mathscr{O}}(N)$.

#### Stationary distribution

Given the transition matrix **T**^(*N* + 1) × (*N* + 1)^, consisting of the transition probabilities given by equations (13) and (14), the stationary probability distribution $\overrightarrow{p}=({p}^{0},{p}^{1},\ldots ,{p}^{N})$ is given by

27 $$\overrightarrow{p}\,{\bf{T}}=\overrightarrow{p},$$

which means it is the left eigenvector of **T** corresponding to the unit eigenvalue^39,65^.

Now rewriting equation (27) yields

28 $$\overrightarrow{p}\,({\bf{T}}-{\bf{I}})={({({\bf{T}}-{\bf{I}})}^{T}{\overrightarrow{p}}^{T})}^{T}=\overrightarrow{0}\mathrm{.}$$

which we can rewrite into the form

29 $${({\bf{T}}-{\bf{I}})}^{T}{\overrightarrow{p}}^{T}={\overrightarrow{0}}^{T}.$$

The matrix (**T** − **I**)^*T*^ is singular, so we use a trick to solve equation (29) for the stationary distribution vector ${\overrightarrow{p}}^{T}$ (see Chapter 2.3.1 in^65^). Setting *p*^0^ = 1 and partitioning the matrix as

30 $${({\bf{T}}-{\bf{I}})}^{T}=(\begin{array}{cc}-\mu  & {\overrightarrow{v}}_{1}^{\,T}\\ {\overrightarrow{v}}_{2} & {\bf{C}}\end{array}),$$

where ${\overrightarrow{v}}_{1}^{\,T}={({T}^{1-},0,\ldots ,0)}^{T}$ and ${\overrightarrow{v}}_{2}=(\mu ,0,\ldots ,0)$. Equation (29) is now equivalent to

31 $$(\begin{array}{cc}-\mu  & {\overrightarrow{v}}_{1}^{\,T}\\ {\overrightarrow{v}}_{2} & {\bf{C}}\end{array})(\begin{array}{c}1\\ {\overrightarrow{p}}^{\ast T}\end{array})={\overrightarrow{0}}^{T}.$$

Solving ${\bf{C}}\,{\overrightarrow{p}}^{\ast T}=-\,{\overrightarrow{v}}_{2}$ yields the solution $(1,{\overrightarrow{p}}^{\ast T})$. Finally, we normalise the vector $(1,{\overrightarrow{p}}^{\ast T})$ to get the stationary distribution ${\overrightarrow{p}}^{T}$. This is given in **Algorithm 9**.

**Algorithm 9:**
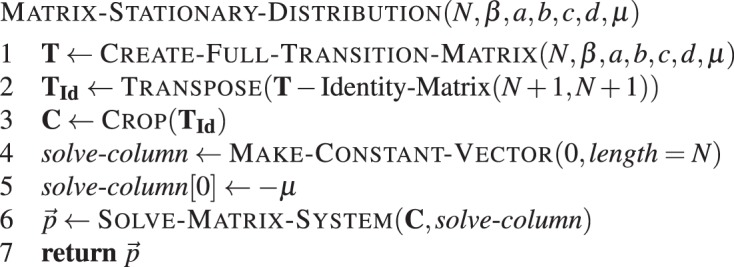
Matrix-based stationary distribution. Stationary distribution for two types in a population of size *N*, with intensity of selection *β*, mutation probability *μ*, and game given by *a*, *b*, *c* and *d*.

Note that we could have omitted the transposition step and constructed *C* directly, but we show it here in the pseudo-code for illustrative purposes.

The normalisation of $\overrightarrow{p}$ can be done in linear time like in **Algorithm 5** for the direct method. As the transition matrix is again a banded matrix with band 1, all operations in this algorithm can be carried out in ${\mathscr{O}}(N)$.

#### Extensions, limitations and scalability

This approach based on the transition matrix is also valid for general Markovian processes that are not birth-death processes. In that case, the transition matrix is not necessarily tridiagonal. For example, this approach works for the Wright-Fisher process in which the transition matrix is dense, but then the computational complexity time is no longer linear, as mentioned above.

The approach described here is not restricted to well-mixed populations. It also applies when considering graphs^7,41^, meaning that the states are not only given by the number of A-strategists, but also by their position on the graph. In this case, there are up to 2^*N*^ states, so the complexity is exponential and turns impractical for larger population sizes. In the most general case of arbitrary intensity of selection, it has been proven that the problem is computationally hard^66^.

The complexity of the computation for structured populations can be alleviated in specific cases, the number of states can be reduced in certain graphs due to symmetry. Also note that in this case the transition matrix is typically not tridiagonal anymore, but it is still sparse^5,41,67–73^. This allows for efficient computational methods which make use of the sparsity structure to store the transition matrix.

In contrast to the analytical method, where only the ratio of transition probability matters, here we have to take the absolute value of the transition probabilities into account. The crux of this method is inverting a matrix. Note that Cramer’s rule implies that every entry of the inverted matrix will be proportional to the inverse of the determinant. When the determinant is either too large or too small, computational errors can arise and need to be addressed. This can be caused by strong selection, just as it happens with the direct method.

Both the direct analytical method and the transition matrix-based approach are based on the Kolmogorov equations of the underlying evolutionary process, i.e. equations (1) and (19). Therefore, for either of the two methods, the computational complexity does not depend on the game. The difference between these two methods arises from the way to solve the equation. The direct analytical solutions are difficult to obtain for general evolutionary processes such as the Moran process in structured populations. The numerical matrix method still works in these cases. Yet, when both methods can be applied, the analytical method needs only *N* − 1 loops as shown in **Algorithm 2**, whereas the numerical method requires more than *N* − 1 loops. In this case, the analytical method is computationally more efficient, cf. Fig. 2a.

Note that a naive implementation of the analytical expression for the fixation probability would lead to a quadratic dependence of the wall time on the population size, see **Algorithm 1**.

Figure 2b shows the wall time for computing the average unconditional and conditional fixation time. The computation of the unconditional and conditional fixation time can be reduced to linear scaling in population size for both methods. By applying smart recursions for the direct calculation and making use of the sparsity of the transition matrix, this reduction from cubic to linear wall time can be achieved. The computation of the conditional fixation time with the matrix-based approach requires computing the fixation probabilities of all states first (see **Algorithm 8**) and this explains why the conditional fixation time takes longer to compute than the unconditional fixation time. The direct method for computing conditional fixation time uses memorisation to store the fixation probabilities, see **Algorithm 4**, which explains why the conditional fixation time also takes longer than the unconditional fixation time for the direct methods.

For computing the stationary distribution, Fig. 2c compares the wall time of the analytical and the matrix-based solutions. They both grow linearly in the population size and the matrix-based approach runs slightly slower. The direct calculation involves a for-loop for the product and keeping track of the sum which is used for a normalisation that is carried out in the second loop. The matrix-based approach applies a trick to avoid the singularity of the matrix, making it possible to solve a simple matrix equation. Using a solver for banded matrices leads to linear wall time as well.

#### Numerical stability

Numerical stability refers to how numerical errors in the input propagate through the algorithm. In a numerically unstable algorithm, errors in the input often lead large errors in the final output. Having compared different algorithms for computational efficiency, it is natural to also perform a comparison for numerical stability. Unfortunately, this is not a trivial task to do formally. Methods abound, but there is no general recipe to determine if a given algorithm is stable or not.

In order to get an idea of stability we use the following method: We use an arbitrary precision system, and, for a given algorithm, set a fixed numerical precision, comparing the relative error arising from precision alone. In a stable algorithm, increasing the precision should lead to smaller errors. Thus, the relative error rapidly decreases, and the computation approaches the true value when precision increases.

For algorithms 2–5 we present these results in Supplementary Fig. S1. The matrix-based algorithms 6–9 are studied in Supplementary Fig. S2. In all cases, increasing precision leads to a rapid decline in relative error. We therefore conclude that our algorithms are stable within the parameter range we analysed already for reasonable numerical precision.

### Simulations

Simulations rely on pseudo-random numbers. Computationally, pseudo-random numbers are often produced by using a deterministic recurrence equation. These are not strictly random but have a number of desirable properties such as having a very long period until they repeat. The series of values generated by this procedure will depend on a seed number that will determine the complete sequence of numbers. This seed can also be used to replicate exact computational runs, for example in debugging simulations.

A number of statistical tests are available to measure the quality of random number generating procedures. In this paper we use the Mersenne Twister algorithm, which has come to be the standard in computational science^74^. This procedure is readily implemented in most common high-level languages. For more details on random number generation we refer the reader to^75^.

Without loss of generality, we assume to have access to a sequence of identically distributed random variables, *U*_1_, *U*_2_, *U*_3_,... uniformly distributed on the unit interval [0, 1]. This sequence is produced by a function RANDOM(). This procedure of generating pseudo-random numbers is ${\mathscr{O}}\mathrm{(1)}$. It is the basis of the simulation algorithm presented below.

Microscopic simulations are based on very low-level descriptions of how individuals interact. We will restrict our attention to games with two players and two types, but generalisations are straightforward. Typically, computing the fixation probability involves only two types, *A* and *B*. In that case, payoffs arising from diadic interactions are given by a 2 × 2 matrix, $$(\begin{array}{cc}a & b\\ c & d\end{array})$$. A process that simulates one step in a Moran process is given in **Algorithm 10**.

**Algorithm 10:**
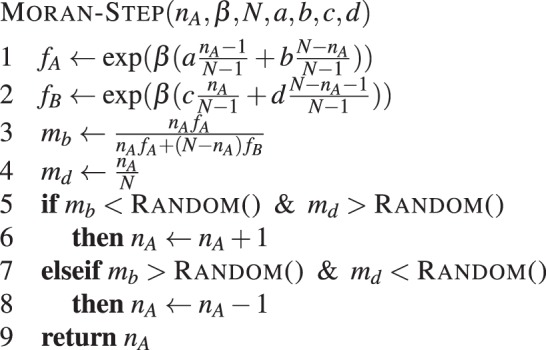
Naive Moran step. Simulates a step of the Moran process with two types in a population of *N* individuals, with intensity of selection *β* and game given by *a*, *b*, *c* and *d* with initial number of type A individuals set to *n*_*A*_.

Lines 1 and 2 determine the fitness of individuals playing strategies *A* and *B* respectively. Note that we use an exponential payoff-to-fitness mapping^25^. Since this is a birth-death process, it is completely determined by two events. A birth event, in which an *A*-strategist is chosen for birth with probability *m*_*b*_ as defined on line 3; and a death event, in which an *A*-strategist is chosen for death with probability *m*_*d*_ as defined on line 4. These probabilities are sampled in lines 5 and 7, and the result is used to update *n*_*A*_ accordingly.

Note that this process can be sped up if we avoid using the procedure in **Algorithm 10** for transitions in which ${n}_{{A}_{t+1}}={n}_{{A}_{t}}$. This happens with probability *k* = *m*_*b*_ · *m*_*d*_ + (1 − *m*_*d*_)(1 − *m*_*b*_). The probability that *n*_*A*_ will increase or decrease in one time-step is thus given by 1 − *k*. Thus, it is possible to estimate the number of time-steps required until ${n}_{{A}_{t+1}}\ne {n}_{{A}_{t}}$ by simulating a geometric distribution with parameter *p* = 1 − *k*. Given that ${n}_{{A}_{t+1}}\ne {n}_{{A}_{t}}$, *n*_*A*_ increases with probability $\frac{{f}_{A}}{{f}_{A}+{f}_{B}}$ or decreases with probability $\frac{{f}_{B}}{{f}_{A}+{f}_{B}}$. This will speed up simulations considerably, by avoiding transitions in which *n*_*A*_ does not change. The resulting procedure is given by **Algorithm 11**. A similar method was used e.g. in^76^.

**Algorithm 11:**
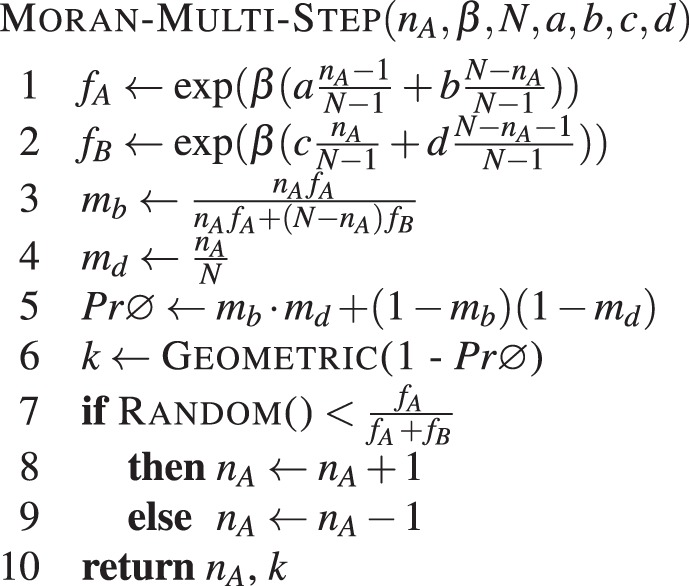
Moran multi step. Simulates several steps of the Moran process with two types in a population of *N* individuals, with intensity of selection *β* and game given by *a*, *b*, *c* and *d* with initial number of type A individuals set to *n*_*A*_.

Here, GEOMETRIC() samples from a geometric distribution. The algorithm returns the value of *n*_*A*_, as well as *k*, which is the number of time-steps required to reach this value.

Simulations work by repeatedly running the MORAN-MULTI-STEP algorithm, which updates the value of *n*_*A*_, and returns the number of evolution time-steps required in the process. Next, we show how to use this algorithm to estimate different quantities of interest using Monte Carlo simulations.

Without loss of generality, we assume that the resident type is *B*, with *N* − 1 individuals in the population. The mutant type is *A*, with a single individual.

The process is determined by the following parameters: *β*, the intensity of selection; *N*, the size of the population; and the payoffs given by *a*, *b*, *c* and *d*.

Since we only care about two types, the state of the population is completely determined by the number of *A*-individuals, *n*_*A*_. We need to run the process in **Algorithm 11** repeatedly, updating *n*_*A*_ until *n*_*A*_ is either 0 or *N*.

The series of random draws required to reach fixation or extinction defines one realisation of the fixation experiment. We can define an indicator random variable *I*, as follows:

$$I=\{\begin{array}{ll}0\, & {\rm{if}}\,{n}_{A}=0\\ 1\, & {\rm{if}}\,{n}_{A}=N\end{array}\mathrm{.}$$

A procedure to simulate a realisation of *I* is given by **Algorithm 12**.

**Algorithm 12:**
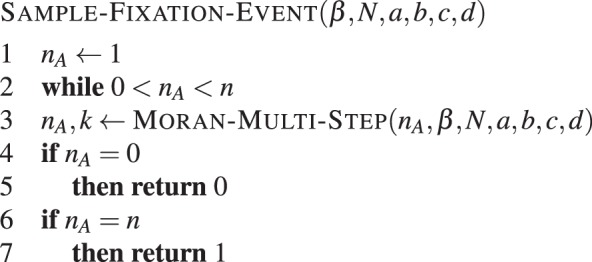
Sample fixation event. Simulates the fixation of type *A* in a population of *N* − 1 individuals of type *B*, with intensity of selection *β* and game given by *a*, *b*, *c* and *d*. Returns 1 if type *A* fixates or 0 if it goes extinct.

#### When to stop the simulation?

The most difficult part of simulating this stochastic process, is finding the right condition to stop the simulation. For simulating fixation probability, we are using an estimate of the variance for coming up with a stopping criterion. This allows using a nice procedure to stop when getting close to the real value, given a target standard deviation. For example, imagine that our goal is to reduce the probability that our estimate for the fixation probability is off by more than *ε* = 10% to less than *δ* = 1%. From *r*_0_ = 100 realisations, we estimate the fixation probability to be ${\bar{X}}_{k}=0.2$, leading to a sample variance of 0.16. As *r*_0_ is smaller than 0.16/(*δε*^2^), we cannot yet be confident that we have enough realisations for a sufficiently good estimate of the fixation probability. Thus, we continue to generate realizations until their number *k* is larger than ${\bar{X}}_{k}\mathrm{(1}-{\bar{X}}_{k})/(\delta {\varepsilon }^{2})$. Once this is fulfilled, the fixation probability is estimated as ${\bar{X}}_{k}$.

As the distribution of the fixation times (conditional and unconditional) is unknown, we cannot use a variance estimate for simulating the fixation time and the stationary distribution. The number of time-steps required to achieve a good estimation is in itself a random variable. Instead, we use an approach based on “moving averages”.

#### Fixation probability

Having defined a procedure to simulate *I*, our task is to estimate *E*(*I*), the expected value of *I*, i.e., the fixation probability. This is equivalent to simulating a Bernoulli random variable, where the success probability is the fixation probability. An estimation ${\bar{X}}_{k}$ is obtained by dividing the number of successful events by the number of Bernoulli trials *k*.

The question that remains is how many realisations, *k*, are needed to provide an estimate that is close enough to the true fixation probability of the process.

Using Chebyshev’s inequality, for a given positive number *ε*, we have

32 $$P\{|{\bar{X}}_{k}-E(I)|\ge \varepsilon \}\le \frac{D(I)}{k{\varepsilon }^{2}},$$

where *D*(*I*) is the variance of the random variable *I*. We want to bound the likelihood with which the estimation deviates from the true fixation probability. Given tolerance value *δ*, then having $\frac{D(I)}{k{\varepsilon }^{2}}\le \delta$ is sufficient. In other words, there should be at least *D*(*I*)/(*ε*^2^*δ*) such realisations. This analysis does not only apply to a Bernoulli random variable, but also holds for other random variables with finite variance.

In particular, for a Bernoulli distribution *I* with parameter *p*, *D*(*I*) = *p*(1 − *p*) = *E*(*I*)(1 − *E*(*I*)). Taking into account that ${\bar{X}}_{k}$ is an estimation of *E*(*I*), the variance of the Bernoulli distribution *D*(*I*) can be estimated by ${\bar{X}}_{k}\mathrm{(1}-{\bar{X}}_{k})$. Thus $k > {\bar{X}}_{k}\mathrm{(1}-{\bar{X}}_{k})/({\varepsilon }^{2}\delta )$ is sufficient to ensure the tolerance. This leads to the following procedure to estimate the fixation probability given a target standard deviation:

1. For the given tolerance parameters *ε* and *δ*, we denote *σ*^2^ = *δε*^2^.
2. Generate *r*_0_ realisations of *I* to compute a first estimate ${\bar{X}}_{k}$. Typically *r*_0_ > 100.
3. Continue to generate additional data, stopping when you have generated *k* values and $k > \frac{{\bar{X}}_{k}\mathrm{(1}-{\bar{X}}_{k})}{{\sigma }^{2}}$.
4. The estimated fixation probability is given by ${\bar{X}}_{k}$, and the number of realisations required is *k*.

The number of events required to estimate a fixation probability *p* has the following form for different values of target *σ*: *R*(*p*) = *r*_0_ + *B*(*σ*)*p*(1 − *p*), where *B*(*σ*) is a function of the target standard deviation^75^. Both *r*_0_ and *B*(*σ*) are independent of the population size. Intuitively, the smaller *σ* is, the larger the realisation number gets, thus *B*(*σ*) should be monotonically decreasing. Furthermore *R*(*p*) ≤ *R*(1/2) = *r*_0_ + *B*(*σ*)/4. Crucially, *R*(1/2) is also independent of the population size.

So far we have only considered the number of sampled events required to estimate fixation. However, each fixation event will take a number of steps that depends on the parameters of the evolutionary process, see also Fig. 1. To account for that, we need to consider the product of the number of realisations and the average fixation time^77^.

To bound the wall time note that *R*(1/2) is independent of *N*. Thus, the wall time to reach a good estimation of the fixation probability is of the same order as the unconditional fixation time.

We note that Chebychev’s inequality is very conservative.

### Fixation times

For fixation time, we describe a procedure to stop simulating based on “moving averages”.

Define a tolerance *ε* = 0.01, a number of initial realisations *r*_0_ = 100 and step size *k* = 100.

1. Generate *r*_0_ realisations of the process and compute the mean (un)conditional fixation time *τ*_start_ of these.
2. Generate *k* more realisations and compute *τ*_next_ as the average of all realisations.
3. While $\frac{|{\tau }_{{\rm{next}}}-{\tau }_{{\rm{start}}}|}{{\tau }_{{\rm{next}}}} > \varepsilon$: set *τ*_start_: = *τ*_next_ and generate *k* more realisations to re-compute *τ*_next_ as the average of all realisations.
4. Return *τ*_next_ as an estimate of the fixation time.

For simulating the conditional fixation time, only the realisations that lead to fixation are saved and the extinct ones are discarded for computing the average. This means that many realisations are wasted, in particular when the fixation probability is low. For the unconditional fixation time, all samples can be used. However, for games with stable internal fixed points, the fixation times can diverge for large population size or strong selection^78^, making it necessary to employ other methods to calculate the time until a meta-stable state is left^79,80^.

### Stationary distribution

In particular for low mutation rates, the system spends a lot of time in the absorbing states. Instead of choosing a random number in each time step (as in our Naive Moran step algorithm 10), we can adapt the procedure developed in our Moran Multi step algorithm 11 for the case of mutations, such that we directly sample the number of times steps until a mutation occurs. For example, when all players are of type *B*, we would have *m*_*b*_ ← *μ* and thus *Pr* ∅ ← 1 − *μ*. In particular for $\mu \ll 1$, this will greatly speed up simulations.

When simulating evolutionary processes to estimate the stationary distribution, we have to find a suitable stopping criterion as there are no absorbing states due to mutation. Therefore, it is important to know for how many time steps the process has to be simulated to achieve a good estimate. Without knowing the resulting stationary distribution, one possibility is to run a certain number of time steps first and then check whether the estimate still changes between time steps (this method does not work accurately for all scenarios, see Fig. 5. To measure the closeness of two distributions, the Kullback-Leibler divergence **D**_**KL**_ is a widely accepted premetric (note that it is not symmetric, so it is important to take care which one is the target distribution and which one is to be tested against the target)^81^. More formally, we consider the following procedure given a target tolerance parameter *ε*:

1. Run the process for *r*_0_ time steps.
2. Define an interval length *k*, a threshold *ε* and set *t* = 1.
3. While **D**_**KL**_[*x*(*t* · *k* + *k*) || *x*(*t* · *k*)] > *ε*:
  run the process for *k* more time steps and increase *t* by one.
4. Return x^*^ as the stationary distribution vector and k^*^ as the stopping time for the procedure.

Here, *x*(*t* · *k*) is the distribution vector of length *N* + 1 when the system is at time step *t* · *k*.

#### Accuracy of the simulation algorithms

**Figure 3:**
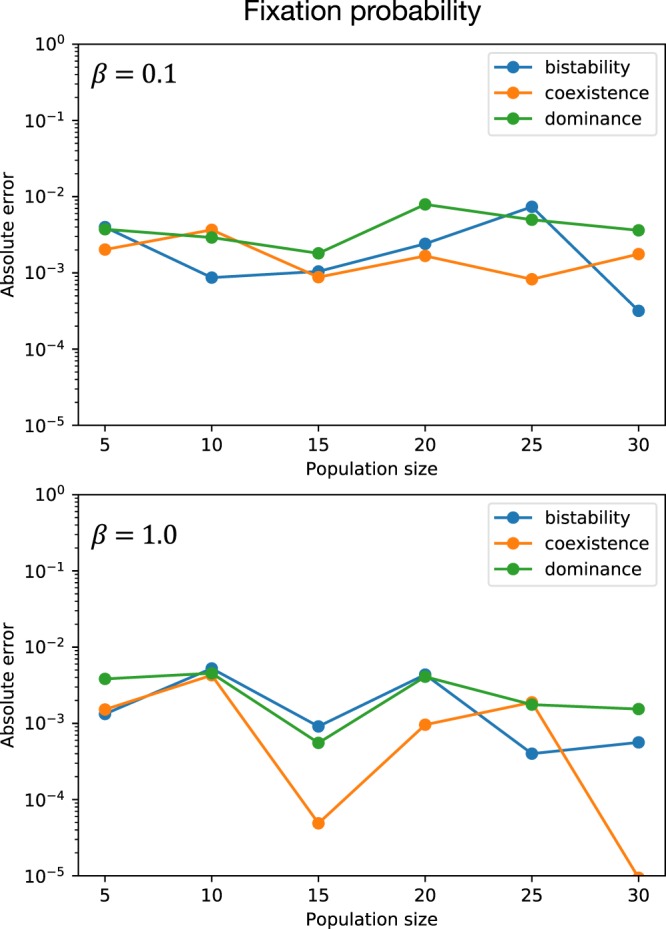
Absolute error of the simulated fixation probability to the target value computed by the direct method. The parameters used for the simulation were *r*_0_ = 100, *ε* = 0.01, *k* = 100 in both panels. Payoffs are $$(\begin{array}{cc}1 & 1\\ 0 & 0\end{array})$$ for dominance game, $$(\begin{array}{cc}0 & 1\\ 1 & 0\end{array})$$ for coexistence game and $$(\begin{array}{cc}1 & 0\\ 0 & 1\end{array})$$ for bistability game. Panel (a) shows results for selection intensity *β* = 0.1 and panel (b) shows *β* = 1.0.

We now study the error of the simulation algorithms. To this end, we compare them to the results of the direct computations. Figure 3 shows the absolute error between the simulated fixation probability and the direct computation.

**Figure 4:**
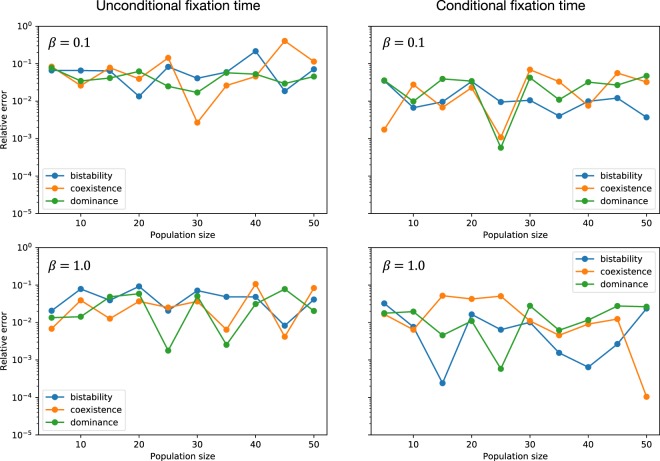
Relative error of the simulated unconditional and conditional fixation time to the target value computed by the direct method. The parameters used for the simulation were *r*_0_ = 100, *ε* = 0.01, *k* = 100 in both panels. Payoffs are $$(\begin{array}{cc}1 & 1\\ 0 & 0\end{array})$$ for dominance game, $$(\begin{array}{cc}0 & 1\\ 1 & 0\end{array})$$ for coexistence game and $$(\begin{array}{cc}1 & 0\\ 0 & 1\end{array})$$ for bistability game. Panels (a) and (b) show results for selection intensity *β* = 0.1 and panels (c) and (d) show *β* = 1.0.

Figure 4 shows the relative error for the simulation method of the conditional fixation time compared to the respective value computed by the direct method. Increasing *r*_0_ and *k* and decreasing *ε* further would result in a decreased relative error, yet this leads to a massive increase of wall time.

**Figure 5:**
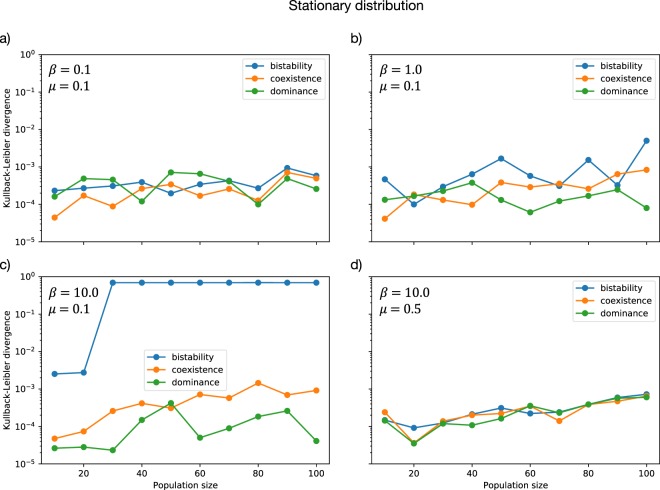
Kullback-Leibler divergence of the simulated stationary distribution to the target stationary distribution computed by the direct method. The parameters used for the simulation were *r*_0_ = 10^2^ · *N*, *ε* = 10^−4^, *k* = 10^5^ · *N* in all panels. Payoffs are $$(\begin{array}{cc}1 & 1\\ 0 & 0\end{array})$$ for dominance game, $$(\begin{array}{cc}0 & 1\\ 1 & 0\end{array})$$ for coexistence game and for $$(\begin{array}{cc}1 & 0\\ 0 & 1\end{array})$$ bistability game. Panels show varying selection intensity *β* and mutation rate *μ*. For strong selection and low mutation rates (**c**), it is difficult to sample the distribution from a single run in the case of bistability, as transitions between the two states are very rare. This explains the large difference between analytical results and simulations in this case.

We measure the accuracy of the simulation algorithm for the stationary distribution by computing the Kullback-Leibler divergence^81^ of the simulated stationary distribution to the one calculated by the direct method. Figure 5 shows that the divergence is rather small for most of the displayed parameters.

Yet for *β* = 10.0 and *μ* = 0.1 the simulation algorithm fares badly for the bistability game. With very high selection intensity, most mass of the distribution is close to the edges. A similar issue arises for very low mutation rate. In our simulation we start from one mutant. Increasing *r*_0_ and *k* can improve the estimate, but for even larger population size and selection intensity this would not help much. To improve the estimate for this case, one should run several simulations starting close to the deterministic interior fixed point or from random initial conditions and then average the resulting distributions.

But this example shows that, in order to spot these issues, it is often advisable to use more than one method.

#### Limitations and scalability

In contrast to the direct and transition matrix-based approaches, for the simulation method, the computational complexity is dependent on the game. The number of realisations is maximal for a fixation probability of 1/2 and this maximum is independent of *N*. Therefore, for any selection intensity, the wall time is bounded from above by a quantity that is proportional to the unconditional fixation time. In particular, under weak selection, it is proportional to the unconditional fixation time.

For these simulation methods, it is important to catch simulations that take too long due to not reaching the stopping criteria within a reasonable time. Especially when algorithms like these are run on clusters, the programmer should make sure to add a counter that stops the simulation at some point.

## Discussion

We have given an overview of widely used methods for computing evolutionary game dynamics in finite populations, see also Table 1. The direct analytical calculation and the transition matrix-based approach are exact methods that rely on a recursive equation–but the results are challenging to interpret and thus an algorithm is needed to numerically assess the results. If implemented accordingly, both methods lead to algorithms that run in linear time, as we have shown for fixation probability, fixation time and stationary distribution. The wall time for both these methods is independent of the underlying game and the selection intensity.

**Table 1:** Overview of the three methods discussed here.

Method	Advantages	Disadvantages
Direct	Wall time is independent of the game	Limited to birth-death processes
	Extendable to other birth-death processes, such as pairwise comparison processes (same complexity)	Not extendable to general graphs
Matrix-based	Wall time is independent of the game	Strongly limited by population size due to size of transition matrix
	Extendable to processes with dense transition matrix, such as Wright-Fisher (increased complexity)	
Simulations	Extendable to Fermi and Wright-Fisher	Wall time depends on the game and the selection intensity
	Extendable to games on graphs and multi-player games	Large number of realisations might be necessary

This table lists their limitations and possible extensions.

The advantage of the matrix-based approach is that it is easily extendable to denser state-spaces than the standard Moran process. Implementing it for the Wright-Fisher process or the Moran process on graphs is straight-forward^41^. But the linear wall time is lost when the transition matrix is no longer tridiagonal, as the efficient special solvers for banded matrices are no longer available in that case.

Simulating the process is quite different from the direct and the matrix-based approach. A large number of realisations has to be carried out to ensure good results. In contrast to the other two methods, the wall time for simulations depends on the game. For example, fixation in games with stable coexistences can take a very long time and averaging over such processes becomes computationally extremely expensive. We have explained how simulations can be sped up by omitting unnecessary time steps where nothing happens. Another, more crucial challenge during simulations is to find the right conditions when to stop. We have provided pseudo-code that illustrates the procedures.

In this way, besides giving an overview of the most widely used computational methods, we hope to provide the necessary tools for applying these techniques to more complex scenarios. Knowing the basics with their limitations and extendibility should help tackling more than two types, multiplayer games, or games on graphs. Evolutionary game theory is applied in various research fields ranging from ecology^82,83^ and cancer^84–86^ to economics and the social sciences^2,87–92^. In all these fields, one usually considers more than two types and efficient algorithms to address the arising computational challenges are important tools.

Analytical methods for evolutionary game theory have been developed substantially in the past few years^12,24,35,58,93–102^. Yet, there will always be a need of computation. Here, we exclusively focussed on fixation probability, average fixation time, and stationary distribution. If one is interested in other observables, e.g. the full distribution of fixation times, other computational methods can be more efficient. For example, to calculate the full distribution of fixation times in a non-iterative fashion, Ashcroft *et al*. have proposed a method that maps a bidirectional random walk into a forward only random walk, which can be used to immediately obtain the full distribution of fixation times^103^. For very large population sizes *N*, however, most computational methods fail–but at least the asymptotic of e.g. the average fixation time can still be assessed^8^. In other cases, a full analysis may not even be necessary. For example, this occurs when mutation rates are small enough such that the process spends most time on very few states^10^. While in the extreme case only absorbing states matter^104^, for slightly higher mutation rates also other, stable states can affect the stationary distribution^105^. Most importantly, such methods can typically even be used for more than two strategies^26,105^.

Whether simulations or analytical approaches are more appropriate depends on the problem at hand. Adami *et al*.^106^ make the case that agent-based simulations should be used for complex and more realistic biological scenarios, and mathematics should be used only to verify the limiting cases that are analytically tractable. In a comment on^106^, Tarnita^107^ argues that new mathematical methods should still be searched for to solve previously mathematically intractable cases. Another comment on this gives an example of a whole set of strategies that can be explored with mathematics, but that would have been invisible to agent-based simulations alone^108^. Thus, mathematics and simulations complement each other. However, for a first exploration and in order to assess dynamics that can often become highly complex or even chaotic even in deterministic evolutionary games^109–113^, it is important to have access to computational methods. Consequently, the development of more efficient algorithms for evolutionary game theory is a growing field of research^66,114,115^.

We have experienced that while tackling a certain question, the chosen method is often secondary as long as it helps towards the solution. This might be because in specific situations, only one method is applicable. But it could also be that the researcher is using their preferred method for historical reasons, which makes sense if switching takes more effort than the benefit the new method yields. By providing a comparison of different methods, we hope to support the work of mathematical and computational evolutionary game theorists re-thinking the use of algorithms.

## Supplementary information

Supplementary Information

## Data Availability

The source code and demo notebooks are available from http://bit.ly/finite_computation_ed.
