# Supplementary material for: Computation and Simulation of Evolutionary Game Dynamics in Finite Populations
Source: Sci Rep. 2019 May 6;9:6946. doi: 10.1038/s41598-019-43102-z (PMC6502801; doi:10.1038/s41598-019-43102-z)
Supplement: Supplementary file 1 — Supplementary Information [file 41598_2019_43102_MOESM1_ESM.pdf]

Supplementary Information

Article: Computation and Simulation of Evolutionary Game Dynamics in Finite Populations
Authors: Laura Hindersin, Bin Wu, Arne Traulsen, Julián García

1 Accuracy of the algorithms

Figures 1 and 2 show the accuracy of the algorithms for direct and matrix-based computations. The procedure is explained in Section Numerical stability. Due to a rapid decline in relative error with increasing precision, we conclude that **Algorithms 2–9** are numerically stable. Figures 2 g) and h) show that the matrix-based approach for computing the stationary distribution is not very stable for small precision. But increasing precision solves this issue. We can also see that **Algorithm 9** struggles with high selection intensity β.

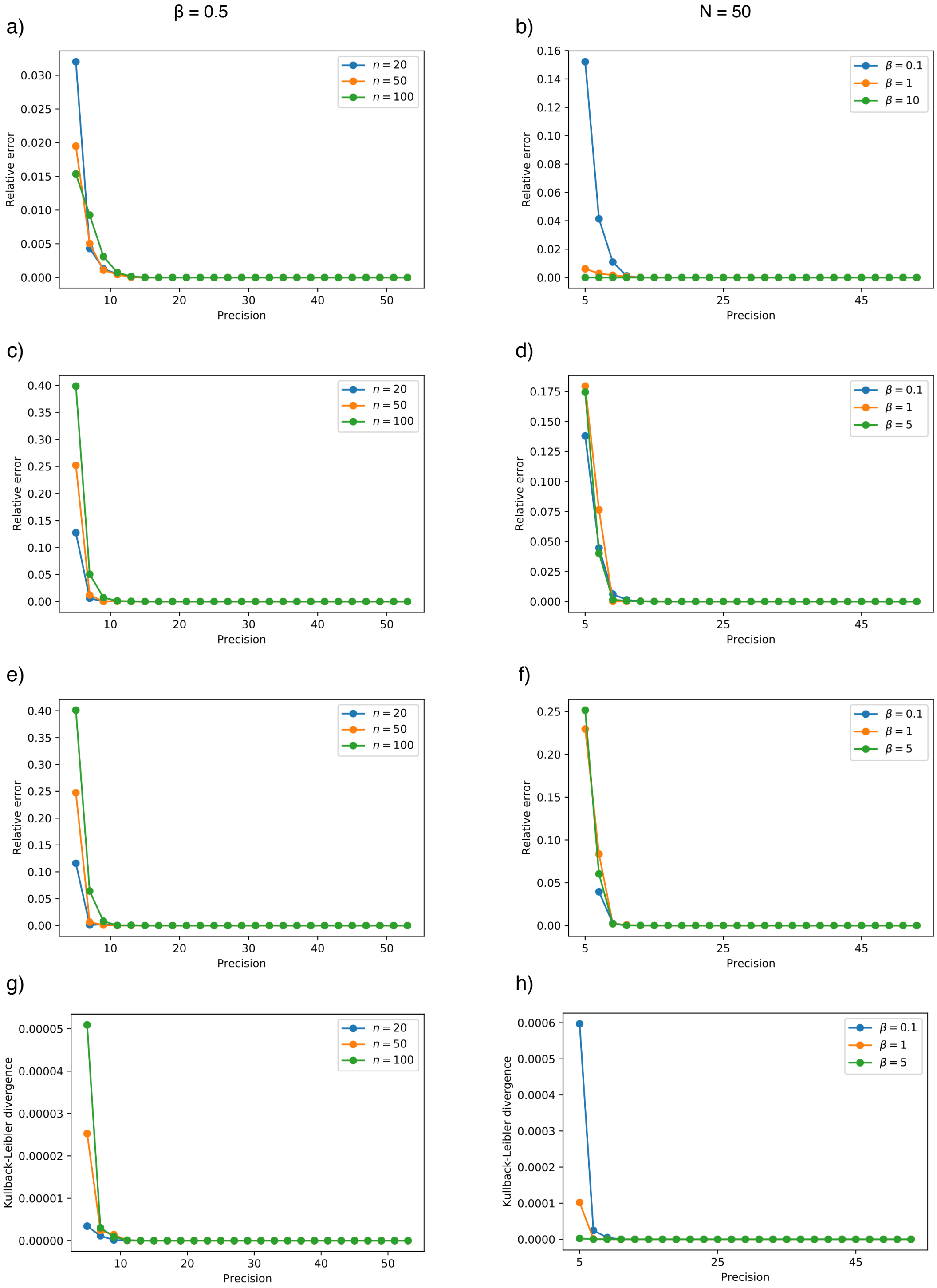

Figure 1: **Accuracy of the direct algorithms for increasing precision.** The first column of panels shows accuracy for fixed selection intensity $\beta = 0.5$ and varying population sizes $N = 20; 50; 100$. The second column of panels is for fixed population size $N = 50$ and varying selection intensities $\beta = 0.1; 1; 5$. Accuracy is measured as relative error for computing fixation probability (a) and b)), unconditional fixation time (c) and d)), conditional fixation time (e) and (f)). For computing the stationary distribution (g) and h)), accuracy is measured as Kullback-Leibler divergence. Payoffs used for all computations were $a = 2, b = 5, c = 1, d = 3$.

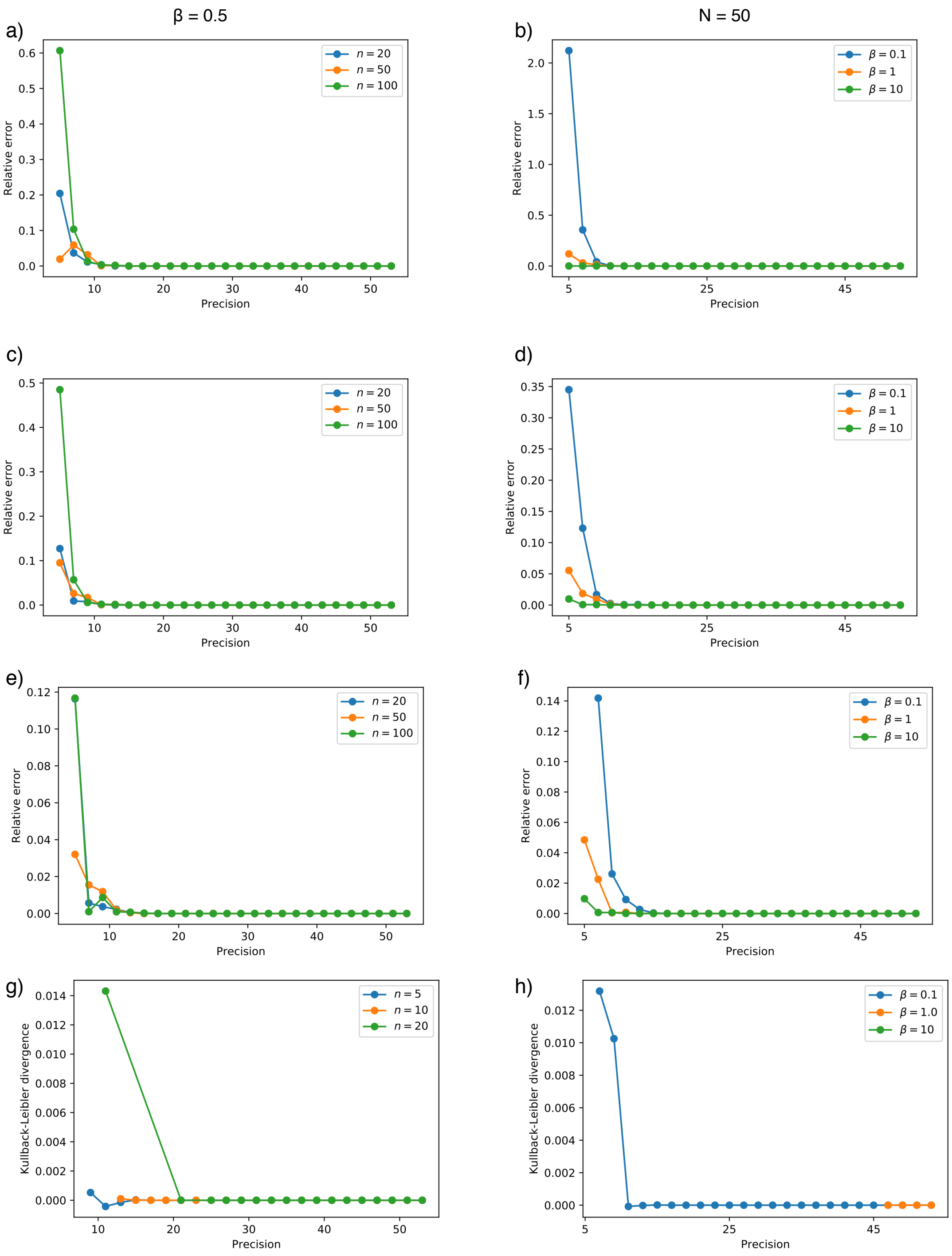

Figure 2: **Accuracy of the matrix-based algorithms for increasing precision.** The first column of panels shows accuracy for fixed selection intensity $\beta = 0.5$ and varying population sizes $N = 20; 50; 100$. The second column of panels is for fixed population size $N = 50$ and varying selection intensities $\beta = 0.1; 1; 5$. Accuracy is measured as relative error for computing fixation probability (a) and b)), unconditional fixation time (c) and d)), conditional fixation time (e) and (f)). For computing the stationary distribution (g) and h)), accuracy is measured as Kullback-Leibler divergence. Payoffs used for all computations were $a = 2, b = 5, c = 1, d = 3$.

2 Weak selection trade-offs

A key quantity in stochastic evolutionary game dynamics is the selection intensity β [1–3]. We are investigating how the selection intensity alters the running time of simulating the fixation probability. As mentioned before, the expected running time is the product of the number of realisations and the expected time of unconditional fixation, i.e., $R(\phi_A(\beta))\tau(\beta)$.

Under weak selection, expanding this to the first order leads to

$$\begin{aligned} R(\phi_A(\beta))\tau(\beta) &= R(\phi_A(0))\tau(0) + \frac{d}{d\beta}R(\phi_A(\beta))\tau(\beta)\bigg|_{\beta=0}\beta + O(\beta^2) \\ &= R(\phi_A(0))\tau(0) + \big(R'(\phi_A(\beta))\tau(\beta) + R(\phi_A(\beta))\tau'(\beta)\big)\big|_{\beta=0}\beta + O(\beta^2). \end{aligned} \quad (1)$$

Here $R(\phi_A(\beta)) = q + B(\sigma)\phi_A(\beta)(1-\phi_A(\beta))$, where q is the basic number of realisations needed. $B(\sigma)$ is a constant based on the accuracy given σ.

Taking into account $\phi_A(0) = 1/N$,

$$\phi_A'(0) = \tfrac{(N-2)a+(2N-1)b-(N+1)c-2(N-2)d}{6N},$$

$\tau(0) = NH_{N-1}$ and

$$\tau'(0) = \tfrac{(Nb-Nd-a+d)N}{2(N-1)}(N+1-2H_N),$$

[4] leads to

$$\begin{aligned} R(\phi_A(\beta))\tau(\beta) &= \left(q + B(\sigma)\frac{N-1}{N^2}\right)NH_{N-1} \\ &+ \beta(B(\sigma)\frac{H_N(N-2)((N-2)a+(2N-1)b-(N+1)c-2(N-2)d)}{6N} \\ &+ \left(q + B(\sigma)\frac{(N-1)}{N^2}\right)\frac{(Nb-Nd-a+d)N}{2(N-1)}(N+1-2H_N)) + \mathcal{O}(\beta^2). \end{aligned} \quad (2)$$

Based on equation (2), the number of Moran steps needed to calculate the fixation probability is given by

$$q(NH_{N-1} + \tfrac{(Nb-Nd-a+d)N^2}{2(N-1)}\beta) + \mathcal{O}(\beta^2)$$

for large population size. This implies that both the zeroth and the first order expansion of the running time are proportional to that of the unconditional fixation time. In other words, the unconditional fixation time determines the Moran steps for simulation under weak selection limit.

3 Calculating the unconditional fixation time

The average unconditional fixation time τ^1 can be calculated by the recursions in equation (5) with boundary conditions $\tau^0 = \tau^N = 0$. It is given by

$$\tau^1 = \phi_A^1 \sum_{k=1}^{N-1}\sum_{l=1}^{k}\frac{1}{T^{l+}}\prod_{m=l+1}^{k}\gamma^m, \quad (3)$$

where $\gamma^m = \frac{T^{m-}}{T^{m+}}$. Taking $\sum_{k=1}^{N-1} \sum_{l=1}^{k} = \sum_{l=1}^{N-1} \sum_{k=l}^{N-1}$ into account yields

$$\tau^1 = \phi_A^1 \sum_{l=1}^{N-1} \sum_{k=l}^{N-1} \frac{1}{T^{l+}} \prod_{m=l+1}^{k} \gamma^m \tag{4}$$

$$= \phi_A^1 \sum_{l=1}^{N-1} \frac{1}{T^{l+}} \underbrace{\sum_{k=l}^{N-1} \left(\prod_{m=l+1}^{k} \gamma^m \right)}_{Q^l}. \tag{5}$$

$$= \phi_A^1 \sum_{l=1}^{N-1} \frac{1}{T^{l+}} Q^l \tag{6}$$

Q^l can be huge for strong selection which leads to an overflow, provided l is small. In other words, for the first few steps, Q^l is huge and then it decreases. Therefore we postpone calculation of large Q^l to later steps and build the sum in a reverse way. Let us introduce $R^l = Q^{N-l}$, then we have

$$\tau^1 = \phi_A^1 \sum_{l=1}^{N-1} \frac{1}{T^{(N-l)+}} R^l. \tag{7}$$

The recursions for R^l are given by

$$R^{l+1} = 1 + \gamma^{N-l} R^l, \tag{8}$$

with $R^1 = 1$.

4 Calculating the conditional fixation time

The average conditional fixation time τ_A^1 can be calculated by the recursions given in equation (9) and boundary conditions $\tau_A^0 = \tau_A^N = 0$. It is given by

$$\tau_A^1 = \sum_{k=1}^{N-1} \sum_{l=1}^{k} \frac{\phi_A^l}{T^{l+}} \prod_{m=l+1}^{k} \gamma^m, \tag{9}$$

where $\gamma^m = \frac{T^{m-}}{T^{m+}}$. Taking $\sum_{k=1}^{N-1} \sum_{l=1}^{k} = \sum_{l=1}^{N-1} \sum_{k=l}^{N-1}$ into account yields

$$\tau_A^1 = \sum_{l=1}^{N-1} \sum_{k=l}^{N-1} \frac{\phi_A^l}{T^{l+}} \prod_{m=l+1}^{k} \gamma^m = \sum_{l=1}^{N-1} \frac{\phi^l}{T^{l+}} \underbrace{\sum_{k=l}^{N-1} \left(\prod_{m=l+1}^{k} \gamma^m \right)}_{Q^l}. \tag{10}$$

Exactly as for the unconditional time, it takes only one loop to calculate the conditional fixation time. Furthermore, we have recursions for Q^l and ϕ_A^l, which saves computation time.

The fixation probability ϕ_A^l is given by [1, 5–7]

$$\phi_A^l = \frac{\sum_{j=0}^{l-1} \prod_{m=1}^{j} \gamma^m}{\sum_{j=0}^{N-1} \prod_{m=1}^{j} \gamma^m}. \tag{11}$$

Thus we have that

$$\phi_A^{l+1} - \phi_A^l = \frac{\prod_{m=1}^{l} \gamma^m}{\sum_{j=0}^{N-1} \prod_{m=1}^{j} \gamma^m} = \phi_A^1 \prod_{m=1}^{l} \gamma^m \tag{12}$$

As for unconditional fixation time, we are reversing the summation. We will again use $R^l = Q^{N-l}$. Let us define $\psi_A^l = \phi_A^{N-l}$ which is the fixation probability of the mutant strategy A when there are $N-l$ such mutants in the beginning. Then we have

$$\tau_A^1 = \sum_{l=1}^{N-1} \frac{\psi_A^l}{T^{(N-l)+}} R^l \tag{13}$$

The following recursions hold

$$\begin{aligned} R^{l+1} &= 1 + \gamma^{N-l} R^l, \quad \text{with } R_1 = 1, \\ \psi_A^h &= \psi_A^{h-1} - \phi_A^1 \left(\prod_{m=1}^{N-h} \gamma^m \right), \quad \text{with } \psi_A^1 = \phi_A^{N-1}. \end{aligned} \tag{14}$$

References

[1] Nowak, M. A., Sasaki, A., Taylor, C. & Fudenberg, D. Emergence of cooperation and evolutionary stability in finite populations. *Nature* **428**, 646–650 (2004).

[2] Wu, B., García, J., Hauert, C. & Traulsen, A. Extrapolating weak selection in evolutionary games. *PLoS Computational Biology* **9**, e1003381 (2013).

[3] Pinheiro, F. L., Santos, F. C. & Pacheco, J. M. How selection pressure changes the nature of social dilemmas in structured populations. *New Journal of Physics* **14**, 073035 (2012).

[4] Altrock, P. M. & Traulsen, A. Fixation times in evolutionary games under weak selection. *New Journal of Physics* **11**, 013012 (2009).

[5] Goel, N. & Richter-Dyn, N. *Stochastic Models in Biology* (Academic Press, New York, 1974).

[6] Antal, T. & Scheuring, I. Fixation of strategies for an evolutionary game in finite populations. *Bulletin of Mathematical Biology* **68**, 1923–1944 (2006).

[7] Traulsen, A. & Hauert, C. Stochastic evolutionary game dynamics. In Schuster, H. G. (ed.) *Reviews of Nonlinear Dynamics and Complexity*, vol. II, 25–61 (Wiley-VCH, Weinheim, 2009).
